# Perfection is a sad and lonely place: A study of existential vulnerability in the life stories of persons struggling with perfectionism

**DOI:** 10.1080/17482631.2023.2219513

**Published:** 2023-06-04

**Authors:** Per-Einar Binder, Vivian Irena Woodfin, Aslak Hjeltnes

**Affiliations:** a Department of Clinical Psychology, University of Bergen, Bergen, Norway; b Department of Clinical Psychology, Solli District Psychiatric Centre, Bergen, Norway

**Keywords:** Perfectionism, narrative identity, qualitative study, existential vulnerability, psychological health

## Abstract

Under what life conditions do individuals turn to perfectionistic striving and ideals as a solution? The present paper examines how people with perfectionism narrate their relationship to our shared existential vulnerability; that we are vulnerable as human beings, and that the ways we relate to this vulnerability have consequences for psychological health. In the present qualitative study, we explored the life narratives told by nine students with perfectionism, drawing on semi-structured life-story interviews. We conducted an explorative-reflexive thematic analysis and identified five themes: 1) Outside—Feeling Alienated, 2) Relating to Chaos, 3) Trying to Control the Painful and Uncontrollable, 4) Islands of Just Being and Positive Contact, and 5) Heading Toward a Balance Between Doing and Being. Their perfectionism can be seen as a way of handling existential vulnerability at a point in their lives where relational resources needed to stay firm in a vulnerable state are lacking. Perfectionistic themes influence their personal identity in the domain of narrative constructions, values, belongingness, and embodiment. Accomplishments were dominant themes in the plots of their narrative self-constructions and values. They felt their “self-made” identities kept others at a distance. However, we also found strivings for a more fulfilling life with broader self-definitions.

## Introduction

How do persons with perfectionism relate to their vulnerability and experiences of the existentially given, undetermined, and uncontrollable aspects of life? Striving towards high standards can be vitalizing and meaningful. Feeling compelled to reach the highest standards, and doing it out of felt necessity, creates suffering. Under what life conditions do individuals turn to perfectionistic striving and ideals as a solution? This paper will examine how people with perfectionism narrate their relationship to an aspect of our common humanity; we are aware of being vulnerable beings.

Our identities are formed both through choices and actions that we control and circumstances and events that are uncontrollable. This intermingling between ambition and vulnerability, of making and being made, can be described as a tragic dimension of the human condition (Nussbaum, 2001). Existential givens make us vulnerable, such as our finitude, our need for meaning and belonging, the groundlessness of our freedom and choices, and our dependence upon our body, with its sensations, emotionality, and capacity for suffering (Binder, 2022a). In this context, the term “existential” refers to the experiential dimension of the concerns and conditions that intrinsically belong to being human. Heather Wallace (2020) connects existential vulnerability with the relational dimension of our being; we are vulnerable because the meaning and value of our actions depend on other people’s responses to us. Robert Stolorow (2016) describes existential vulnerability as painful possibilities that define our existence and that loom as constant threats, such as injury, illness, death, and loss. Hanne Laceulle (2017) uses the term existential vulnerability to address a broader range of vulnerabilities that cannot be remediated because they intrinsically belong to the human condition, such as our finitude and the undetermined aspects of choices that bring us purpose and meaning. This paper will use the term in this broad sense, addressing the vulnerability due to non-negotiable existential givens. Irvin Yalom (1980) describes such fundamental aspects of human existence as “ultimate concerns” that are always potentially anxiety-provoking and painful. When they feel overwhelming, ultimate concerns will be involved in an “existential psychodynamic” where they are put away from awareness through psychological defences.

While the existential vulnerability is part of common humanity, situational vulnerabilities specific to personal, cultural, or social situations vary. Mackenzie et al. (2014) describe pathogenic vulnerabilities «as a subset of situational vulnerabilities” that refers to varieties of situational influences that are troublesome in a moral sense. “Erich Fromm (1947) operates with a similar distinction between existential dilemmas and historical contradictions. In line with Laceulle (2017), in this paper, we will use the term “contingent vulnerability” to describe both situational, historical, and pathogenic vulnerabilities, and for our purpose—those that are related to the development of perfectionism.

Perfectionism was initially studied in clinical contexts where it was mostly thought to be a character trait with a defensive function. Karen Horney (1950) regarded one possible source of perfectionism to be unrealistically high and often authoritarian parental demands through childhood, and the resulting feelings of worthlessness, shame, and guilt. However, most fundamentally, “the child does not develop a feeling of belonging or we,” something that awakens a “basic anxiety.” Perfectionism, therefore, arises both as a defensive strategy to keep overwhelmingly painful feelings at a distance and as a compensatory strategy for a deeply rooted experience of being defective. Horney (1950) described the phenomenology of this type of perfectionism clear and vividly:

He holds before his soul his image of perfection and unconsciously tells himself: Forget about the disgraceful creature you actually are; this is how you should be; and to be this idealized self is all that matters. You should be able to endure everything, to understand everything, to like everybody, to always be productive.

(p. 65)

According to Horney, this type of perfectionism is a strategy with high costs. It stimulates self-hate, and it drains the person for energy and resources. It alienates the person from their “real self,” basic needs, healthy growth, and last, but not least—they are alienated from relating to others with the spontaneity of their real feelings. Horney’s theory is quite similar to Alfred Adler (1938/1998, who describes certain types of perfectionism as a compensatory strategy for an underlying “inferiority complex.” Adler is also explicit about a healthy, purposeful, and adaptive drive towards “perfection” as a fundamental motivational force in human life. However, the type of perfection Adler spoke about was not striving towards perfect performance. Instead, it was the will to overcome hindrances, and strivings for competence, wholeness, and completion, not only for oneself but also for the common good of humanity (Watts, 2012). Searching for high-level goals can also be healthy; it depends upon whether it has its origin a sense of belonging, is freely chosen and flexible, or is compensatory, self-centred, and feels forced.

From a psychometric point of view, perfectionism can be operationalized as a multidimensional personality trait, which consists of unrealistically high expectations and overly critical self-evaluations (Frost et al., 1990; Woodfin et al., 2020). The two main categories of perfectionism are also described as “perfectionistic strivings” and “perfectionistic concerns”: Strivings are features associated with personal standards (typically described as high, exceptionally high, or unrealistically high), whereas perfectionistic concerns are features associated with harsh self-evaluation and doubts about self-worth (fears, feelings of inadequacy and negative reactions) (Stoeber & Otto, 2006).

Egan et al. (2011) argued that perfectionism is a transdiagnostic process because it is elevated across numerous anxiety disorders, depression, eating disorders, increases the risk of eating disorders, and is a maintaining factor across disorders. On the background of this distinction between strivings and concerns, it has been proposed that perfectionistic strivings also can be a “healthy” form of perfectionism. However, it can also be argued that healthy ambitions and strivings towards excellence do not mainly have perfection as a goal but are motivated by prosocial values and feelings of personal and relational purpose (Greenspon, 2000). Although perfectionistic concerns is the category most strongly associated with higher psychopathology outcomes, perfectionistic strivings are also so, although to a lesser degree (Limburg et al., 2017).

Perfectionism can be internally and externally motivated (Stoeber et al., 2009). Self-oriented perfectionism is internally motivated and related to high personal standards. Socially prescribed perfectionism is externally motivated and associated with the experience of high expectations from others. Socially prescribed perfectionism is a maladaptive form of perfectionism associated with adjustment problems and psychological distress. Although stressful, self-oriented perfectionism may have positive aspects, such as conscientiousness and higher performance.

Is perfectionism on the rise due to the increasingly competitive individualistic culture in modern capitalistic societies? In the 1930s, Karen Horney (1939) stated that “A compulsive perfectionism would strike no one as a problem in a rigidly puritanical group” (p. 428). Although religious puritanism has lost its force, some aspect of its work ethic is still alive. In achievement-oriented societies, high personal standards easily become a norm. As shown in a study conducted by Curran and Hill (2017), there has been an increase in perfectionistic traits among young people since 1989, causing higher stress levels. The authors conclude that their findings indicate that in recent generations, young people perceive others as more demanding of them; they perceive themselves to be more demanding of others and are more demanding of themselves. Curran and Hill (2017) argue that three broader cultural changes may explain these shifts: 1) the emergence of neoliberalism and competitive individualism, 2) the rise of the doctrine of meritocracy, and 3) increasingly anxious and controlling parental practices. They highlight that the rise of neoliberalism has led to a cultural movement from collectivism to a new culture of competitive individualism. They argue that “increasing levels of perfectionism might be considered symptomatic of how young people are coping—to feel safe, connected, and of worth—in neoliberalism’s new culture of competitive individualism» (p. 413). They also note that the rise of meritocracy has increased the need for individuals to strive, perform, and achieve in schools, universities, and the workplace to compete for the resources to a socially successful and financially stable life. Meritocracy has increasingly become an organizing principle for how we think about ourselves and other people (Sandel, 2020). These broader cultural pressures have also influenced the family structures and parental practices.

Flett et al. (2002) propose several pathways towards developing perfectionism, among them controlling parenting, social expectations and social learning, the social reaction to a harsh environment, and anxious rearing. Curran et al. (2017) also highlight conditional parental regard as a cause for perfectionism. They have examined both what can be described as self-critical perfectionism, associated with depressive tendencies, and narcissistic perfectionism, associated with grandiosity and promoting an idealized self-image. Parental conditional regard was a powerful predictor of self-critical perfectionism. Curran et al. (2017) propose that continued exposure to conditional parental regard strengthens the association between contingencies of self-worth, guilt, and shame. The resulting may be harsh self-evaluative tendencies that are indicative of self-critical perfectionism. On the other hand, narcissistic perfectionism seems to have other causes, such as overindulgence in childhood, social modelling, and parents’ use of their children to fulfill their ambitions.

Perfectionism may also play a role in forming personal identity (Negru‐Subtirica et al., 2021). Self-oriented perfectionism is associated with a strong commitment to values, future plans, and goals. This might foster a sense of responsibility for the self of the future that can be adaptive in a highly individualistic, competitive, and meritocratic society. On the other hand, people with socially prescribed perfectionism tend to overanalyze through rumination and worry about core personal values and future plans. From a psychodynamic perspective, perfectionism will imply a split in self-experience and personal identity between an idealized “perfect” self and an underlying sense of shame and worthlessness (Chen et al., 2015; Horney, 1950).

Ricoeur (1991) describes life as “an activity and a passion in search of a narrative” (p. 29). Narratives have a fundamental role in giving shape and meaning to personal identity. Narratives organize life’s temporal structure, with a past, present and future. They bring the otherwise chaotic episodes of our lives into orderly patterns to make self-reflection and autobiographic reasoning possible (Habermas & Reese, 2015). Narrative identities must both create an experience of order and sameness and at the same time be flexible enough to handle change, which also is a fundamental aspect of the human condition (Ricoeur, 1991). Narrative identity, therefore, may be defined as a person’s internalized and evolving story of the self (McAdams, 2011). Dan McAdams (2008) has developed a life-story interview as an instrument to explore key scenes, characters, trends, and themes with particular relevance to personal identity.

In a mixed-methods study applying life story interviews, perfectionists were found to have a high frequency of agency themes, especially related to winning and/or achieving heightened status (Mackinnon et al., 2013). In a thematic analysis of life story interviews, Farmer et al. (2017) found relationship success and gratitude most central to adaptive perfectionists and agenting redemption most central to maladaptive ones. Maladaptive perfectionists often had life themes connected to adverse life events and a tendency to strive for self-improvement to cope with those events. In their narratives, they tended to focus on improving themselves rather than reaching out to others for help when trying to make sense of the adverse events in their lives.

**Figure 1.**
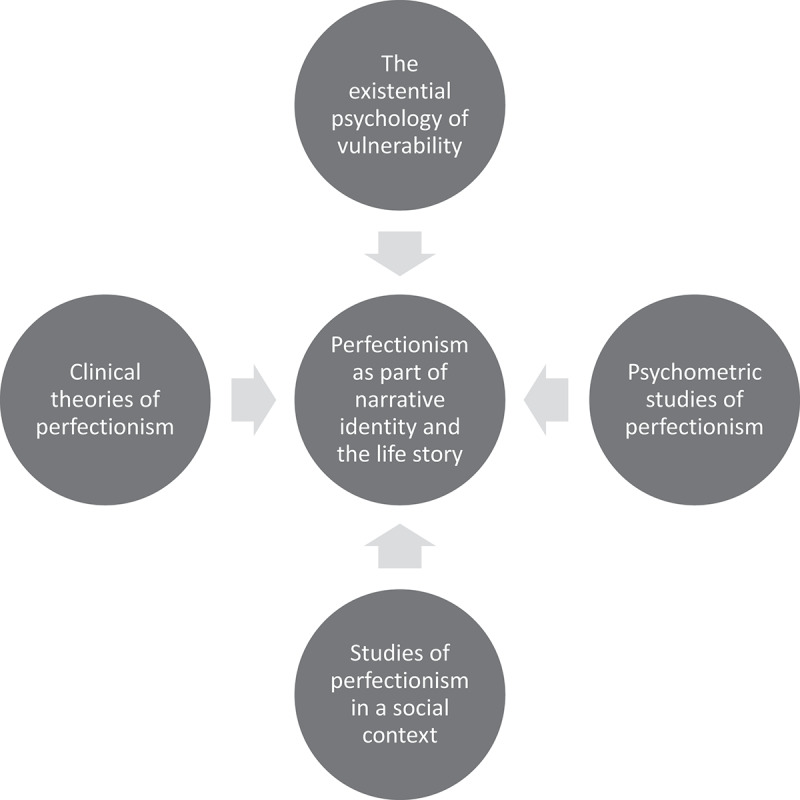
An overview of theoretical approaches to perfectionism.

We have provided an overview of the theoretical approaches we apply to understand and analyse perfectionism we use in this study in Figure 1.

The material that we describe and analyse in this article is part of a larger study exploring both the possibility to change maladaptive perfectionism through a self-compassion intervention (Woodfin, Molde, et al., 2021) and the phenomenology of perfectionism in the life stories of a sample of highly perfectionistic individuals. In a previous paper, we examined how individuals in this sample understand the relationship between painful experiences and how they relate to others (Woodfin, Hjeltnes, et al., 2021). Narratives are a fundament for personal identity and, at the same time, a means to reflect upon and give meaning to other aspects of identity, such as values, belonging, and embodiment (P.-E. Binder, 2022a). This paper will present and discuss the results from that part of life story interviews that address how these persons relate to existential vulnerability on a broader level.

The aim of this paper is to explore how persons with perfectionism relate to existentially given, undetermined and uncontrollable aspects of life. Self-definition and identity can always be challenged—and sometimes hindered—by losses, loneliness and experiences that become overwhelming on an outer and inner level. More specifically, we address the following question: How do persons with high perfectionism relate to existential vulnerabilities through their life stories?

## Materials and methods

### Participants

A total of nine individuals (two male, seven female) were recruited from a larger sample (*N* = 388) of students who signed up for a self-compassion course for students struggling with perfectionism. The course was targeted at students who wanted to learn self-kindness through mindfulness and self-compassion. We selected participants that scored in the top 5% on the Norwegian translation of the Frost Multidimensional Perfectionism Scale-Brief (FMPS-B) (Burgess et al., 2016; Frost et al., 1990; Woodfin et al., 2020). The top 5% is not a formal cut-off, but we selected participants with these very high scores in order to explore life stories of students that we with good reason could assume had very high levels of perfectionism.

The FMPS-B scale has been translated and back-translated by a second individual in order to confirm that the translation reflected the measure’s original intended meaning as outlined by the World Health Organization’s translation process guidelines for forward translation and back-translation (World Health Organization [WHO], 2020). The scale was validated in a Norwegian sample and the Cronbach’s α coefficient shows good internal consistency (α = 0.83) (Woodfin, Binder & Molde, 2020). FMPS-B consists of a total of eight questions, with two subscales comprising of four items each—“Evaluative concerns” (e.g., If I fail at work/school, I am a failure as a person) and “Strivings” (e.g., I set higher goals for myself than other people).

The participants’ ages ranged from 20–50 years. In total, ten individuals were invited to be interviewed, and nine agreed to participate. The email invitation stated that we were interested in understanding how perfectionistic individuals tell their life stories. The second author conducted the interviews, who would hold the self-compassion course they had signed up for together with the first author. The participants were informed about this. The researchers had no prior roles to the participants. Participants were not provided any incentives for participating in the interview, and participants were informed that participation in the course was not contingent upon participating in the interview.

### Procedure

All participants were interviewed at the Faculty of Psychology, University of Bergen. Interviews varied in length, between 120 and 360 min. Each interview was conducted over the course of one or two days depending on length. The participants varied in their story-telling style, rhythm, and verbal fluency. We therefore allowed for the interviews to last as long that each participant felt was needed to explore their life stories. All participants wanted to complete the entire interview.

We used a semi-structured life-story interview developed by McAdams (2008), which was translated to Norwegian. These interviews aimed to gain a greater understanding of how students with a high degree of perfectionism constructed their narrative identity and, through this, how they related to and reflected upon their lived experiences.

### Interview protocol

The original English version of the life story interview is available online (McAdams, 2008). The first author translated this interview guide into Norwegian. The second author, a native English speaker who also speaks Norwegian fluently, confirmed that all meaning has been captured in the translation.

The interviewer first asked the interviewees to give an overview of their lives by thinking about their life as if it was a book or novel and dividing it into “chapters” and providing a brief plot summary for each. Next, the interviewee is asked to focus on a few key events that stand out as especially memorable or important in the life story, a high point, low point, turning point, high point in childhood, the low point in childhood, and a series of other scenes that are notable for their emotional significance, or their meaning for the life course. For each scene, the interviewee is asked to describe when and what happened at the moment, who was there, what they were thinking and feeling, how the scene was ultimately resolved. They were also prompted to reflect upon what they think the scene says about them as a person and insights about their life story that might be derived from the scene.

### Data analysis

We chose a qualitative method that retains the thematic content of the interviews to trace subtle nuances in how the participants make meaning through their life stories (Braun & Clarke, 2006, 2019). This explorative-reflexive thematic analysis was conducted with hermeneutical-phenomenology as an epistemological premise (P.-E. Binder et al., 2012). The commitment to, and preparation for, exploring the participants’ concrete lived experience, with an awareness of its symbolically mediated pre-narrative quality, is the basis of the hermeneutic phenomenological element in our approach (Ricoeur, 1991). In line with the hermeneutic ideal in reflexive methodology, we used our dialog with the participants’ narratives to increase our awareness of taken-for-granted assumptions that we bring with us in our reading of them and reflect upon the interpretive lenses our professional and theoretical backgrounds bring in. Our goal was to establish a reflexive stance towards preconceptions that might affect the way the interviews were conducted as well as our analysis of the transcribed material (Alvesson & Sköldberg, 2000; Finlay, 2003). In line with usual practice in reflexive methodology, we present information about our professional background in the “researcher”-section that might be relevant for how the focus of the study is formed and for how data is interpreted. Due to the interpretive nature of narratives, the study of narratives is an interactional enterprise in which the findings are products of the interplay between researchers and participants.

**Figure 2.**
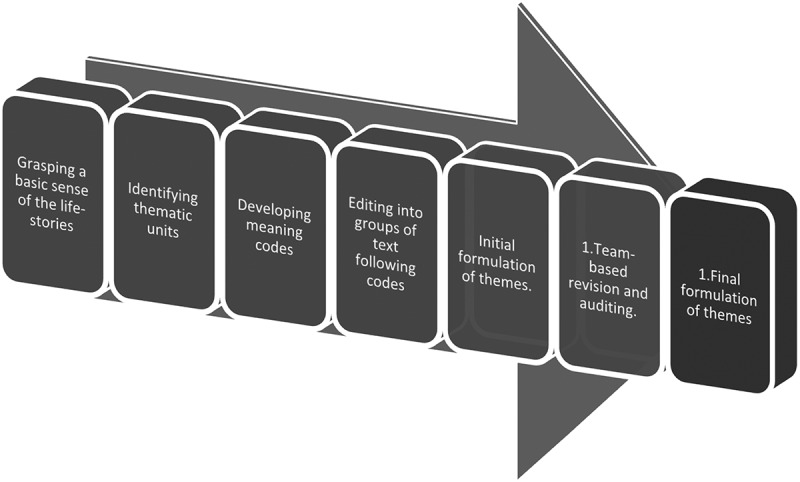
Steps in the analysis.

Analysis proceeded through the following steps (Figure 2):

1. All researchers read all the transcribed material to obtain a basic sense of each of the participants’ life stories, both through emphatic introspection of the particular episodes and to grasp the temporal structure of the story as a whole. Gradual recognition of personal and professional preconceptions was also part of this phase.
2. Examining those parts of the text relevant to the research question, the first author identified separable thematic units representing different aspects of the participants’ narratives.
3. The first author developed “meaning codes” for those units, a concept or keywords attached to a text segment to permit its later retrieval (Kvale & Brinkmann, 2009).
4. Then, he edited the text following those codes into groups of text, with the assistance of Nvivo 12 software (QSR, 2020).
5. The first author interpreted and summarized the meaning within each of the coded groups of text fragments into conceptions and overall descriptions of meaning patterns and themes reflecting what, according to his understanding, emerged as the most critical aspects of how the participants related to existential vulnerability.
6. All three authors turned back to the overall text to check whether voices and points of view should be added, could develop the conceptions and descriptions of themes further, or represented correctives to the preliminary line of interpretation. The third author, who was not part of the team that conducted the self-compassion course, had a leading role in critically auditing the identification of thematic units and meaning patterns.

“Meaning patterns” appears when we condense and sum up the content of meaning units of relevance for our research question and compare the experiences and plots inherent in the narratives of several participants (P.-E. Binder et al., 2012). A pattern emerges when there is a convergence between the narratives of different participants and a moderate degree of divergence between them, making the pattern thematically rich. We express the narrative content of a meaning pattern by formulating themes. In this way, identifying a “meaning pattern” combines the hermeneutic element of interpretation with the phenomenological element of commitment to the participants’ lived experience and empathic use of imagination.

### Researchers

The study was conducted within the Research Group for Clinical Psychology at the University of Bergen. The first author is a professor in Clinical Psychology with 27 years of clinical experience, training in experiential, psychodynamic, and mindfulness-based therapy, broad experience with qualitative research methods, and interest in existential approaches within theoretical and philosophical psychology. The second author is a research fellow and psychologist with seven years of clinical experience and training in self-compassion and mindfulness-based approaches. The third author is an associate professor in clinical psychology with 12 years of clinical experience and an interest in humanistic, experiential, existential, and relational approaches to psychotherapy.

### Ethical considerations

The Regional Committee approved the study for Medical and Health Research Ethics, Region North. All interviewees were given pseudonyms, and identifying information was changed to preserve anonymity. All participants provided written informed consent prior to participation.

The interview was conducted in a sound-isolated room with only the researcher and participant present to ensure confidentiality and the participant’s privacy. The participants were informed that the audio recordings would be deleted after the interviews were transcribed and that the transcriptions would be anonymized. They were informed that participation in the project was voluntary and that they could withdraw their consent at any time without giving a reason and without negative consequences for their participation in the self-compassion course. If they withdrew their consent, their interviews would not be used in any further research.

## Results

In our analysis of these life stories, an overarching theme was the deep-rooted feeling of alienation that arose from overwhelming, painful, and often emotionally traumatic events. Most of the participants also described their lives as chaotic at some point. In the chapters of their lives following the arrival of alienation and chaos, the participants described past and present lives dominated by the need to seek perfection to achieve control, structure, and to some degree, self-worth and purpose. However, perfection and a sense of control do not relieve the underlying pain. Most of them describe the best moments of their lives as not dominated by the need to achieve and accomplish. They rather described them as moments of just “being,” either in relationships to others or through feeling connected to nature and surroundings. They harboured hope that their accomplishments would have a relational meaning and a balance between doing and being for the future chapters of their lives. We identified five main themes in our analysis: 1) Outside—Feeling Alienated, 2) Relating to Chaos, 3) Trying to Control the Painful And Uncontrollable, 4) Islands of Just Being and Positive Contact and 5) Heading Toward a Balance Between Doing and Being. We will describe these thematic findings in more detail below.

### Outside – feeling alienated

A common theme in all these narratives was the experience of events in childhood or adolescence coming out of control in a way that left them feeling alienated. In critical moments and phases of their life, they felt not properly belonging and living on the fringe or outside of the community of others. The events that led them there varied; those were stories of being left alone, bullied, or isolated because of unfortunate life circumstances, such as physical or mental illness. However, there were strong similarities in the mental states that these events awoke in them: they felt not properly at home in their worlds.

For Thomas, the experience of alienation first occurred after a huge conflict with his stepmom as a teenager, with some cruel false accusations towards him. He says that “I had no network. I lost a lot of what had been my sense of safety”. He lost the possibility to influence how his family viewed him. The way his stepmother interpreted his actions and intentions made him feel alienated from his family.

Dana became an orphan as a teenager after traumatic and overwhelming events. She also experienced bullying at junior-high school. Dana then started to feel that “It was never safe to be myself around other people because one never knew what they would use against me or use to hurt me.” She describes that she has turned away from others to protect herself. In some sense, this type of protection has worked; Dana says that she has “never been hurt in a relationship.” However, the price has been high. She feels like an outsider; “I have never been good enough, but that I feel I must,” and she struggles to compensate, “I always feel that I should be better, better than all others somehow.” At a general level, Dana describes feeling “isolated from the world.”

Also, for Paula, the ways others viewed and interpreted her actions felt threatening. She describes herself as having been a timid and lonely child; “sitting alone during recess, and I could not understand why nobody wanted to be with me. And I did not dare to ask anyone”. She also became very self-aware and feared that others would discover how lonely she was; “the fear that someone could discover that I was sitting alone, in a negative way. That they would think that ‘Oh, look at her, she sits there alone”. She dressed in black and listened to punk rock as a teenager. Paula struggled with depression and felt she was living outside the community of peers. It felt like “living in a bubble” and like “not having a life.”

Mostly, the experience of being an outsider or different was frightening. For many participants, it was also connected with feelings of shame and a sense of living without a purpose. However, in a couple of life stories, this was a more ambivalent theme, partly connected to experiences of being exceptional.

Katie describes that she was “so far ahead of others” that school became boring. She came from a small town where the other pupils did not care about school as much as her. She says that the teachers “put her above the others” and tried to use her as an inspiration and sometimes even a co-teacher for the other children. She was ambivalent about this and felt that she fell outside the class community into a “strange” position. She says that “they did not invite me to party at junior high because they feared that I would look down on them.” Today she studies law and feels partially socially integrated, but not very close to her fellow students; “I do not want them to know certain things about me, in case they are unreliable and tell others, or our friendship ends. I do not really trust them”. She longs for others to like her but doubts that they will: “do they really want me here, or is it just that I am useful for them?”.

### Relating to chaos

Periods of chaos, or a constant feeling of threatening chaos, were common in most of these life stories. Together with the experience of living outside, this also made feelings of meaninglessness and futility a constant threat. Chaos made them vulnerable in an unbearing way.

Both for John and other participants, chaos was strongly related to feeling alienated. For him, chaos resulted from destructive relational events and conflicts at home as a teenager. Mother and father separated, came back to each other, then separated, came back, then, at the same time as his girlfriend’s father died, it came out that his mother had an affair. He says, “It felt scary at home and difficult to be there. I became passive and lazy for a while. It was chaotic”. For John, the relationship between chaos and order is either-or: “It must be 100%, and it must be the right chronology, or it is nothing. When there is chaos, I do not have any overview; there are no results, nothing to show”.

Also for Agnes, this relationship between order and chaos was dichotomous. When she talks about a period of her life where she was living alone, feeling lonely and depressed, she describes that:

“I had no control. I felt that my life was terrible and felt shameful for it. I think that the contrast says a lot about me. When I do not manage to do things, when I could not go to school because it felt like too much, then everything falls apart. It may explain that I am an all-or-nothing type of person. I must have it all; if not, I might as well give it all up”.

In Agnes’s story, as well as in the stories of most of the other participants, chaos was unwanted and felt uncanny. However, there were two interesting exceptions: In Paula’s and Susie’s stories, experiencing chaos and some loss of control also was a theme connected to a creative lack of order. Chaos made them vulnerable, but at certain points, they felt the courage to face this vulnerability. For them, this sense of chaos signalled the possibility of something new in their life arriving. Paula describes this positive lack of control in connection with the story of becoming a student at an international college:

“Now something happens. It was not I that decided that change was going to happen; it happened by itself. It felt like a pleasant tingling in my stomach. Tingling of joy … It was an experience of being out of control. It was a joy but a little tense at the same time. It was like entering something unknown, just like before you are going for a journey”.

Susie also talks about a critical phase in her life where positive changes happened associated with a positive lack of control. She had a year after high school and before college, where she worked in the USA. The new surroundings and flux of new people in her life felt like a possibility for another life and a different persona:

“I had a totally different life here that I had over there. It feels like different persons living different lives. That’s because I lived a very different life in the US, and I had very different people around me, some of whom left before I left, new people arrived … A flux of people, and a lot of excitement”.

Although with high scores on perfectionism, Paula and Susie had a strong storyline in their narratives free from the either-or and all-or-nothing relationship between order and chaos that characterized the other participants.

### Trying to control the painful and uncontrollable

Seeking perfect results at school can be a way of building structure and finding agency in a painful and chaotic life. As shown in the “relating to chaos”-theme, perfection can be experienced as an antidote to chaos. However, perfectionism is a “solution” that only addresses the chaos, not the pain related to being vulnerable, and therefore it will never be enough to bring balance in their lives. In their life stories, the participants talked about a struggle for control that they failed in. That resulted in a sense of hopelessness. With a part of themselves, they realize that it is a struggle that they could not ever succeed.

John describes that he always planned how a project he made for himself at school should turn out. He got agitated on the inside when they did not but hid it, thinking that others could not understand how he felt about it, “oh God, they do not understand anything.” It felt impossible to explain to others that he constantly needed things to be 100%. With one part of himself, he knew that things could not be just as he planned, but it felt like “when I had started, I could not let go of it.”

In Agnes’ life story, the need for control appeared after a period of illness as a child “I was ill as a child, long periods in the hospital before I was six. I do not remember much, but it has made an impact, for instance how my parents looked at me”. The painful aspect of this storyline seems related to both treatment at the hospital and the fact that the parents could not offer her a protective shield from these overwhelming experiences. A sense of inner chaos gradually gave rise to a strong need for control:

“During the years after that, I built up a lot of anxiety and a need for control. Everything had to be perfect. I realized it when I was 14. It felt like a lump, very compact, all difficult experiences, all my fears, and it was like a compact mass. I didn’t really know what was there. However, I knew that it had always been there”.

Dana describes how her need for control arose in a situation where she perceived chaos and lack of control in her relational life. She is also aware that the underlying theme is the uncontrollable aspects of life; “for me, the perfectionism has been very much about a lot of things that I cannot control. But I pick out things that I can control, such as being competent at work, and how others look at me”. However, in her narrative, it becomes clear that neither her skills at work, nor her social persona, are things that Dana feels that she can control: “I never feel good enough, but I just feel that I have to be.” She tells about this struggle in a very ambivalent way, sometimes believing, sometimes doubting the value of her attempts for perfection. She says, “The only things that I can control are my thoughts and actions, thus, how I am as a person in the world.” However, being a person among others also means entering an ambiguous terrain. Dana often ends up “feeling disappointed about myself, because I am not better, that I am not that competent” and that she has “such weak moments, such negative qualities.” She reflects upon her ways of thinking and sees that she perhaps has higher standards than others: “I have the ideal version of myself, and when I differ from that, I become very disappointed. I see that it is quite natural and human, but it feels like a defeat for me. I have made this ideal version of myself, and it very much dictates how I am as a person. And then I easily enter a downward spiral when I don’t succeed”.

### Islands of just being and positive contact

Although experiences of alienation and painful struggles for self-worth were dominant themes in the participants’ life stories, they also describe episodes and brief chapters that stand out in stark contrast. If we conceptualize perfectionist strivings as a doing-theme, these moments can be regarded as a being-theme. In a troubled sea, they were islands of just being. They could let down their guard. These could be moments spent in surroundings that awoke feelings of peacefulness. It could also be more intense and joyful moments of positive contact. The experiences always also had a highly significant relational frame, even when they were sometimes mostly associated with particular physical surroundings.

John tells about visiting his grandparents in their cottage when he was a child as experiences that gave a significant contrast to the rest of his life. It was a glimpse into another way of living:

“It was really quite simple, that wonderful summer I traveled with my grandparents to their cottage and lived together with them there. It was something very specific, this place, not very fancy, primitive in a way, and incredibly idyllic. It has always been this place; it has always been this peacefulness; there has always been this cottage. When I was a child, we were always there in the summer. And when we arrived, we were always there, in that mode. Everything else was irrelevant. To go there, with them, was very good. Then I had a very good time, very relaxed, not disturbed by worries in any way”.

The story about this place almost has an awe awakening quality, like a glimpse into an idyllic eternity. When asked to reflect upon the experience, John thinks it reflects his need for a mental space where he can retreat from busyness and relax.

Agnes talks about an important moment of being-mode from her late teenage years. She was an exchange student in Australia, sitting on the beach with two very good friends. Agnes remembers that she realized this was something extraordinary; “Wow, can really life be this good!”. When she could connect with these positive emotions, she experienced a deep sense of freedom. Agnes felt that “happiness is important in itself, and what happened before and after did not really play a role. I was free and felt safe within myself”. When asked to reflect upon this episode, Agnes thinks it made her aware that she “needed something different from what I usually thought I needed. To enjoy the silence”. It also made her reflect upon her perfectionism and gave her distance to it: “Perhaps I am not that much the most-competent-and-productive-and-best student, successful girl, that I always had thought that I am.” Rather, that there was more to whom she felt she was than her achievements.

In Katie’s life story, she shares about a holiday when she was four years old as a significant contrast: “We sat in the car, driving through Denmark. I remember that we sang my favourite song and that I ate candy that they bought for me at the boat. And I thought that this is nice”. What was important to her was that “Mom and dad enjoyed it, my brother enjoyed it, and I enjoyed it. It was happy and peaceful. All of us were happy. It is a happy memory. My happiest memory”. When asked to reflect upon the episode, Katie thinks that “Perhaps it says that I do not need very much to become happy. The happiness of others makes me happy”.

For Laila, an episode with her cat when she was seven also represents safety and peace. She says that “it was summer, and it was a very nice evening. I sat in our garden and talked with my cat. And I had this feeling that it was safe, it was a holiday, no scary things would happen the next days”. Animals have always meant something special for Laila. She says that “it feels a little bit embarrassing to say; It has always been easier for me to get contact with animals than humans.” With animals, she can let her guard down: “You can trust animals, they are honest, they understand. My cat was my best friend. She was number one”. When she reflects upon the episode, she thinks it tells very much about her need for safety. In addition to the contact with her cat, she also felt a vital connection to the surroundings and her sensory experience: “It was something with the mood; the light was nice, it was green, it had been a very nice day, it was sunny—when I was a child, I had this ability to be easily carried away by things around me.”

For Lilly, experiences in nature combined with positive relational experiences with her dad are an essential contrast to her perfectionist strivings. She says, “I have so many memories from the mountains and walking in the forests with dad … it was a lot of trees, view towards the mountain and deer. I felt safe”. When asked to reflect upon the episode, she says, “That is so long since last time I was in such a situation. Mostly I am in my head, in another place … but on these walks, I was just there”. She describes it as a solid contrast to her present situation, something she longs for: “Often I wish I was there, it was very nice, now everything is chaos.”

### Heading toward a balance between doing and being

The participants’ narratives about the following chapters in their lives, and the plot they make about hopes for the future, contain components of “being”-themes in addition to ambitions connected to achievements. They strive for success. However, they would like to do so in a way where more than achievements and perfect results matter. They dream of a life where strivings could have a relational frame or some other kind of higher value that adds a sense of purpose and meaning.

John dreams about a future that seems aligned with his positive childhood memory of being in the cottage with his grandparents. He says, “I hope to build a secure base, have someone to share thoughts with. To do things that I like, cultivate these activities”. John describes an adventurous side of himself that wants to travel, perhaps sailing. However, he also addresses that he has another side of himself that seeks a successful career and money and wonders how to balance that.

Agnes wants a future where she makes a positive difference in other people’s lives, helps them, and inspires them. She thinks this would also “make my own life easier to master.” She wants “a more peaceful life, a life with much more safety.” Agnes will not give up her perfectionistic striving but hope that she can “master or use my need for control and the achieving side of myself in a way that becomes an advantage.” She thinks that this will be possible “if I find a purpose that is larger than myself, and that at the same time is possible to master as an individual.” This reflects a shift from her high-level compensatory goals to strivings from a sense of belonging and purpose.

Laila first mentions her dream of marrying her boyfriend and having a family. Then she says, “it feels a little bit embarrassing to say, but I want to get a Ph.D.” She says that “if I could study all my life, I would do so.” Although her life as a student has much stress connected to her perfectionistic strivings, studying also has a deeper purpose to her. She says that “the higher reason is that I love to learn, I love to understand things, and that feeling you get after having struggled for a while and things fall into place.”

Susie also brings a positive attitude to the unknown when it comes to her view on the future. She says that: “I hope that I will continue to find out things about myself and that I have more realizations where I think that now I feel something that I have not felt before. That I surprise myself, again and again”. More specifically, she also dreams about a job, to be able to buy an apartment, having a lot of good friends, and settling down with a partner. However, the process seems as essential as the results; “to be free and experience something new and be more fearless. If I do that, I will surprise myself and find out what I want”.

## Discussion

The context of the interviews was that the participants had signed up for a self-compassion course for students who wanted to treat themselves with more compassion, mindfulness, and kindness. They were aware of their feelings of isolation and pain. They were also aware that their need for perfection, to some extent, had taken control of their life and did not lead them to a place that they wanted to go. As they were help-seeking individuals, the sample therefore may consist of participants who tend to regard their perfectionism as a problem, and it seems reasonable to assume that they are at a point in their life where they are seeking personal change.

A core theme connected to existential vulnerability in these life stories was that participants struggled with the undetermined nature of human relationships, especially that the way others interpret their actions and relate to them is outside the range of possible control. This undetermined aspect of human relationships becomes threatening through overwhelming or traumatic events that became turning points in their life stories, for some also involving the death of loved ones or illness. A sense of alienation appeared. Important moments or periods in their life become chaotic. The feeling of being protected and at home in their world hit cracks. Through Heidegger’s (1927/1996) conceptualizations, this can be described as an experience of the “unhomely” or “unheimlich”; the experience of an undisturbed everyday world of relationships and involvement in goal-related tasks is disrupted. When the taken for granted intelligibility of the world is lost, anxiety arises. This anxiety can be understood as a specific emotion colouring the experience of the everyday world, as described by Joseph Sandler (1960) when he conceptualises how trauma can change the ordinary experience of a “background of safety” into a “background of the uncanny” . Anxiety in this context can also be understood as an existential state that shows the “groundlessness” of being and indicate that choices of a new direction in life are necessary (Heidegger, 1927/1996; Kierkegaard, (1844/2013). However, when this overwhelming feeling of alienation occurred, the participants were not in a position where constructive choices could easily be taken. Our previous analysis also showed that lack of trust and distancing from others were central aspects of their experience (Woodfin, Hjeltnes, et al., 2021).

Rejection, unreliable others, illness, and traumatic loss are in themselves contingent and situational types of vulnerabilities. As Fuchs (2013) describes, certain life circumstances can make persons especially vulnerable to life’s “boundary situations,” such as death and hazard, and make persons retract from the suffering associated with them.

Stolorow (2016) points out how personal emotional trauma can bring us face to face without our common existential vulnerability. The specific contingent vulnerabilities in a person’s life point towards vulnerabilities shared by all humans. For example, an interpersonal conflict and rejection point towards the existential reality that life is both fundamentally relational and, simultaneously, parts of ourselves and our lived experience are out of reach from others and therefore isolated (Binder, 2022b; Yalom, 1980). In the same way, the death of loved ones points towards the reality of finitude in human existence.

In their narratives, participants describe how they felt forced to achieve a superficial sense of meaning through accomplishments with a perfectionistic quality. Their perfectionism can be seen as a way of handling existential vulnerability at a point in their lives where relational resources needed to stay firm in a vulnerable state were lacking. In line with Yalom’s (1980) existential psychodynamics, their perfectionism can be seen as a defensive strategy. A sense of being unworthy, for some of them even doubt their right to exist, must be disconfirmed by being extraordinary. Sometimes this results in some victories of gratifying but temporary successes. However, feelings of restlessness, tiredness, and threat of isolation and futility dominate their inner world.

These findings are in line with the increasing attention given to the role of early adversity in the development of perfectionism (Chen et al., 2019). Adverse childhood experiences and parenting behaviours foster feelings of despair, shame, defectiveness, powerlessness, and felt insecurity. Exposure to childhood adversity has been found to be associated with elevated socially prescribed perfectionism and non-display of imperfection. Childhood experience of abuse has been found to be a unique predictor of socially prescribed perfectionism and perfectionistic self-presentation styles. Moreover, childhood experience of family dysfunction is positively associated with non-display of imperfection. Perfectionistic tendencies and behaviours may be a means to seek order in a chaotic environment and regain a sense of personal control following traumatic or uncontrollable events, and an attempt to avoid similar experiences recur. Although driven by needs for acceptance and belonging, persons high in perfectionistic traits might act in ways that tend to disconnect them from others (Hewitt et al., 2017). An underlying feeling of defectiveness and unworthiness gives rise to sensitivity for rejection, which leads to perfectionistic self-presentation that others can experience as “cold” and distant. It seems reasonable to assume that this disconnection might be especially psychologically toxic in combination with emotional trauma, as trauma often leads to alienation deriving from a sense of living in a reality different from that inhabited by others (Stolorow, 2015).

Being confronted with existential givens also gives possibilities for growth and heightened awareness of possible choices and priorities in life. As Stolorow (2015) points out, a sense of belonging is essential for such growth: “Vulnerability that finds a hospitable relational home could be seamlessly and constitutively integrated into whom we experience ourselves as being” (p. 136). In life stories, painful and even traumatic and unbearingly painful events can be “turning points” that make a new sense of belonging, growth, and new choices possible (McAdams & Bowman, 2001; Pals & McAdams, 2004)

Islands of “just being” and powerful experiences of positive contact with others were also distinctive elements in these life stories. These moments, present in all the participants’ stories, were exceptions to the main plot. Nevertheless, at the same time, these experiences were something that they could pick up as themes for the next chapter in their life stories: Their hopes for their future. In their hopes for the next chapters in life, the strive for perfection was still there. However, they dreamt of a future where they and their accomplishments mattered for others. A history of neglect is associated with a feeling of not mattering (Flett et al., 2016). On the other hand, developing a felt sense of being important and feeling significant to others build resilience in young people (Flett, 2018)

It struck us how the participants also had a non-dominant but nevertheless strong storyline of a healthy self, ranging from the past and into their possible futures. These can be described as forward-edge strivings: viable “tendrils” of healthy childhood motivations, expectations, and hopes of getting what is needed in the future (Tolpin, 2002). We do not know the outcomes of these specific participants in the self-compassion course. However, the fact that they signed up for the course might also be part of these forward edge strivings towards healthy ways to relate to vulnerability. In general, the eighty-nine participants that completed the intervention showed reductions in maladaptive perfectionism, depression, and anxiety and increased self-compassion as measured by the FMPS-B, Major Depression Inventory, State-Trait Anxiety Inventory and Self-Compassion Scale—Short (Woodfin, Molde, et al., 2021)

In our sample, perfectionism is related to existentially given, undetermined, and uncontrollable aspects of life in a way that formed and shaped their self-definitions and, thereby, their personal identity. The role of existential vulnerability in personal identity can be explored both within the domains of narrative constructions of self, values, belonging, and embodiment.

### Narrative constructions

The ability to make new forms of meaning and narrative order makes it possible to relate to existential dread and “groundlessness” on an ontological level (Heidegger, 1996; Ricoeur, 1991). In the participants’ narratives, the undetermined aspects of relationships and overwhelming and often traumatic experiences threatened their need for coherence and continuity in their life stories. Stories of accomplishments as a means to gain control over a chaotic outer and inner world were dominant in these self-definitions. In some sense, giving up accomplishments would mean giving up themselves. At the same time, at the point in their lives where these participants were interviewed, another subdominant but more hopeful self-narrative about more “being” and less “doing” in their life had the quality of “work in progress.”

### Values

In their lives so far, and in their current life situations, accomplishments have become a value in itself for their participants. As pointed out in the literature on identity development, the process of exploring possible life values, making choices of values, and committing to them are building blocks for personal identity (Berzonsky, 2004; Erikson, 1968). The “Islands of Just Being and Positive Contact” and the “Heading Toward a Balance Between Doing and Being” themes point towards exploring new possible values. They hoped that one day perhaps, their accomplishments could matter to others. They hoped they could change their life situation so that accomplishments did not have to be at the centre of their identity. They were searching for a life where communion could be a value that holds that centre. However, they felt that such a commitment could make them too vulnerable.

### Belonging

As Yalom (1980) points out, there is a substantial psychological and existential difference between feeling left alone and willingly seeking solitude to stay present and tune into one’s own way of experiencing the world. For the participants, aloneness, for the most part, meant loneliness and a feeling of alienation. Important relationships have proven unreliable through rejection or losses, as we examine in the “Outside—Feeling Alienated”-theme. The vulnerability connected to taking a leap towards close relationships often felt threatening. As a reaction to this, the participants tended to describe a “self-made” identity where they held others at a distance. This is also in line with Hewett and Flett’s (2017) perfectionism social disconnection model, which points out how perfectionistic self-presentation, although driven by inordinate needs for acceptance, easily results in others’ negative reactions, with alienating social disconnection as a result.

### Embodiment

In line with Winnicott (1960) and Stern (2018), an embodied sense of continuity or “going on being” can be regarded as a fundament for personal identity. The participants often describe a sense of inner chaos and the experience that feelings can overwhelm them. In this way, embodied and emotional experience poses a threat and makes them feel vulnerable. The one-sided weight given to achievements, and the intellectual activity connected with this, put emotional needs at a distance. It gives them some sense of continuity in their self-experience but simultaneously makes them feel alienated in their own lives. The experience of “just being” that they miss, and some of them explicitly long for, can be seen as an embodied type of being that could offer continuity on a more fundamental level.

In all these four domains of personal identity, we found strivings for a more fulfilling life with a stronger fundament for their self-definition. It appeared as an inner call for a freer and more generous way of being towards themselves and others. This can also be understood as a sense of responsibility or being accountable for oneself, to oneself, that also is described as “existential guilt” (Binder, 2022c). They have a feeling that they owe themselves more than being the perfect one.

### Scope and limitations

These life-story interviews were conducted in a context where participants had a) signed up for a self-compassion course, and b) this course was announced to be targeted at students that wanted to be more self-compassionate and mindful. Therefore, it seems reasonable to assume that these participants may be motivated to change some patterns in their lives or are contemplating doing so. This motivation may partly explain why “forward edge”-strivings, hope, and visions for a different future became central in their narratives. The number of participants in this study is limited. This makes an in-depth exploration of their life stories possible but gives reason for caution regarding generalizability. Further studies of the relationship between perfectionism and existential vulnerabilities, especially in other life situations, will make more general conclusions possible.

## Conclusion

On the background of the life narratives analysed in this study, perfectionism can be seen as a way of handling existential vulnerability at a point in life where relational resources needed to stay firm in a vulnerable state are lacking. We can understand perfectionism as part of existential psychodynamics, as an attempt to control uncontrollable and anxiety-provoking aspects of the human condition. At the same time, in these life narratives, perfectionism needs to be understood in the context of specific life conditions with overwhelming and traumatic events leading to experiences of loneliness and alienation. Perfection and a sense of control did not relieve the underlying pain. Most participants describe the best moments of their lives as not dominated by the need to achieve and accomplish, but as moments of just “being. They told about possible and hoped-for future chapters of their life stories where accomplishments would have a relational meaning and a balance between doing and being. The life-story interviews were conducted in a context where participants had signed up for a self-compassion course, and these participants may be more motivated to change patterns in their lives than other groups of students with perfectionism. However, the life stories of these participants highlight existential dilemmas related to perfectionistic strivings that may be relevant specifically for help-seeking students with perfectionism.
